# Unlocking the Hidden Potential of *Agave tequilana* for the Green Synthesis of Antibacterial ZnO Nanomaterials: A Waste-to-Value Nanotechnology Approach

**DOI:** 10.3390/ijms262311545

**Published:** 2025-11-28

**Authors:** Ghulam Mustafa Channa, Atiya Bhatti, Juan G. Sotelo, Sergio Obregón, Eugenio Sánchez-Arreola, Jorge L. Mejía-Méndez, Diego E. Navarro-López, Edgar R. López-Mena, Angélica Lizeth Sánchez-López, Luis Marcelo Lozano

**Affiliations:** 1 Tecnologico de Monterrey, Escuela de Ingeniería y Ciencias, Av. Gral. Ramón Corona No 2514, Colonia Nuevo México, Zapopan 45121, Mexico; gmchanna139@gmail.com (G.M.C.); atiya.bhatti10@gmail.com (A.B.); edgarl@tec.mx (E.R.L.-M.); alsl@tec.mx (A.L.S.-L.); 2 Institute of Advanced Materials for Sustainable Manufacturing, Tecnologico de Monterrey, Santiago de Querétaro 76130, Mexico; jgsotelof@tec.mx; 3 Facultad de Ciencias Físico Matemáticas, Universidad Autónoma de Nuevo León, Cd. Universitaria, San Nicolas de los Garza 66455, Mexico; sergio.obregonal@uanl.edu.mx; 4 Departamento de Ciencias Químico Biológicas, Universidad de las Américas Puebla, Santa Catarina Mártir s/n, San Andrés Cholula 72810, Mexico; 5 Tecnologico de Monterrey, Escuela de Ingeniería y Ciencias, Epigmenio González 500, San Pablo, Santiago de Querétaro 76130, Mexico

**Keywords:** zinc oxide, nanoparticles, phyto-assisted synthesis, antibacterial activity, phytochemicals, agricultural-waste, sustainable nanotechnology

## Abstract

Traditional nanoparticle synthesis methods often rely on hazardous chemicals, raising concerns about their environmental impact. This study reports the green synthesis of zinc oxide (ZnO) nanoparticles using aqueous extracts from three distinct parts of *Agave tequilana*: the stalk (ZnO-S), heart (ZnO-H), and leaves (ZnO-L). The aim was to explore the influence of the different plant parts, each with their respective phytochemical profile, on the structural, optical, and antibacterial properties of the resulting nanoparticles. The synthesized ZnO-NPs were extensively characterized using UV–Vis spectroscopy, ATR-FTIR, X-ray diffraction (XRD), field emission scanning electron microscopy (FESEM), and energy-dispersive X-ray spectroscopy (EDS). The results revealed that ZnO-S exhibited the smallest particle size (~18.3 nm), the highest crystallinity, and the most uniform morphology. Optical analysis showed bandgap energies of 3.13 eV (ZnO-S), 2.99 eV (ZnO-H), and 3.02 eV (ZnO-L), with ZnO-S demonstrating enhanced UV absorption and reactive oxygen species (ROS) generation potential. Antibacterial assays against *Staphylococcus aureus* and *Escherichia coli* confirmed strong bactericidal activity for all samples, with ZnO-S showing the largest inhibition zones, approaching the efficacy of the reference antibiotic kanamycin. This work highlights the fundamental roles of plant-derived phytochemicals as natural reducing and capping agents and emphasizes the valorization of agave stalk and leaves, traditionally treated as agricultural waste for cost-effective and eco-friendly nanomaterial production. The findings reveal the untapped potential of *Agave tequilana* as a sustainable source for high-performance nanomaterials, paving the way for green innovations in antimicrobial and environmental applications.

## 1. Introduction

Nanotechnology is a rapidly advancing field of modern research with wide-ranging applications across science and technology. It enables the fabrication of novel materials at the nanoscale level, characterized by unique features such as morphology, size, and distribution [1,2]. Nanoparticles (NPs) can be synthesized via physical, chemical, or biological approaches. Physical methods, such as pulsed laser deposition, sputtering, molecular beam epitaxy, and thermal evaporation, typically require high energy input, elevated temperatures, and a pressure system [3,4,5]. Chemical approaches, including sol–gel, hydrothermal, solvothermal, spray pyrolysis, and microwave-assisted synthesis, offer greater control over NPs properties [4,6]. Nonetheless, both physical and chemical synthesis methodologies possess inherent limitations. Physical methods force substantial energy input, increased temperatures, and pressures, leading to time-consuming procedures, whereas chemical approaches utilize harmful substances that may pose a threat to the environment [6,7]. Consequently, green synthesis methods have emerged as an ideal alternative due to their environmental sustainability, cost-effectiveness, and exclusion of hazardous chemicals [8,9]. These methods are safe, non-toxic, and enable facile, one-step synthesis with control over NPs’ size and morphology [10]. Among various metal oxide (MO) nanoparticles, zinc oxide nanoparticles (ZnO-NPs) have received significant attention owing to their versatile functionalities, affordability, and biocompatibility with organisms at the molecular level [11,12].

ZnO-NPs synthesized via green methods exhibit physicochemical, biodegradable, and biocompatible properties, making them suitable for a broad range of applications [13,14], in which antibacterial, antifungal, antiviral, antioxidant, antidiabetic, and/or wound-healing activities are of great relevance [15,16,17]. Such characteristics of ZnO-NPs, coupled with their versatile optical, catalytic, and mechanical properties, make them integral to advanced materials science to address critical healthcare challenges and drive sustainable technological innovations across the industrial and biomedical sectors [18]. The optical, electrical, and catalytic properties of ZnO-NPs can be finely tuned by modifying their particle size, doping with other elements, or altering their crystalline structure [19]. These tunable features influence essential performance characteristics, including surface area, bandgap energies, and photocatalytic characteristics [20]. Enhanced properties, such as ROS (reactive oxygen species) generation, make ZnO-NPs highly effective against pathogens and valuable in healthcare settings [21,22].

In green synthesis, plant-derived phytochemicals such as polyphenols, flavonoids, terpenoids, tannins, phenolic, and carboxylic acids act as potent reducing and capping agents [23,24,25]. These bioactive compounds facilitate the chelation and reduction of metal ions, e.g., Zn^2+^, forming coordination intermediates that convert into ZnO upon calcination [26]. According to some published works, the diverse functional groups in phytochemicals play a key role in particle stabilization, control over geometry, and eco-friendly synthesis conditions [23,27,28]. The success of this method depends almost entirely on the specific phytochemical profiles of the plant sources used. Numerous plant-based studies have demonstrated the successful synthesis of ZnO-NPs using extracts from leaves, flowers, seeds, pulp, or aerial parts of different species, as summarized in Table 1. Furthermore, some works have reported the production of composite ZnO-based materials, such as Ag/ZnO [29], ZnO/MgO [30], or Cu_x_O/ZnO [31] through phytoextract-mediated processes. However, the potential of using different structural parts from a single plant source remains largely unexplored.

**Figure 1:**
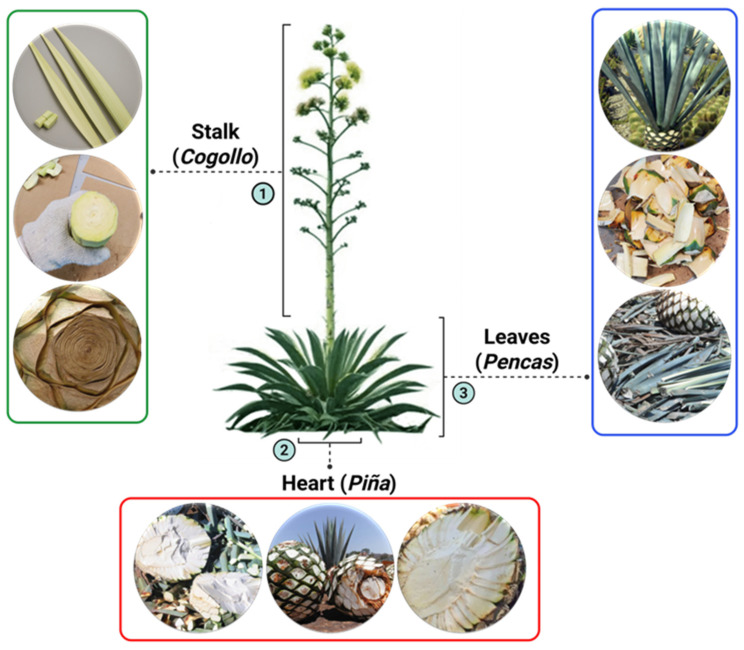
The various parts of the *Agave tequilana* plant utilized in the extraction process for the synthesis of ZnO-NPs.

The present research aims to comparatively evaluate ZnO nanoparticles synthesized from extracts derived from three different plant parts, namely the Stalk (Cogollo), Heart (Piña), and Leaves (Pencas) of *Agave tequilana*, a plant locally known as Agave Azul in Mexico (see Figure 1). *Agave tequilana* belongs to the family *Asparagaceae*, subfamily *Agavoideae*, and is native to several Mexican states, including Jalisco, Colima, Nayarit, Michoacan, and Aguascalientes [55]. It flourishes in semi-arid climates at an altitude above 1500 m (~5000 feet) and grows well in sandy, nutrient-rich soils. This rosette-forming succulent contains spiky, fleshy leaves, which can grow over 2 m (~7 feet) in length [56]. *Agave tequilana* stands as a vital economic driver in Jalisco, Mexico, renowned worldwide as the foundation of Tequila production and a hallmark of regional identity. From the *Agave tequilana* plant, three parts can be identified: the stalk, the heart, and the leaves.

The stalk, locally known as cogollo, is often regarded as waste and is usually removed at an early stage to ensure that the heart (piña) maintains the highest concentration of sugars essential to high yields in Tequila production. Regardless of this, it presents a unique opportunity for sustainable repurposing. Its rich chemical composition and underexplored properties make it a fascinating candidate for innovative applications. Utilizing the stalk not only reduces environmental waste but also offers exciting possibilities for experimental research, bridging the gap between conventional practices and modern sustainability-driven exploration. The heart, or piña, as farmers also know it, is rich in agavins-branched oligosaccharides, predominantly composed of fructose [57,58], emphasizing its industrial and economic significance, which makes it a commercially valuable resource and a key factor in its suitability for the production of Tequila. The leaves, or pencas, are traditionally removed and discarded during Tequila production, as they play no significant role in the beverage’s processing. Instead, these leaves have long been valued in rural communities for their lasting fibers. They have been cleverly used in artisanal crafts to create essential items such as ropes, nets, bags, sacks, and mats.

**Figure 2:**
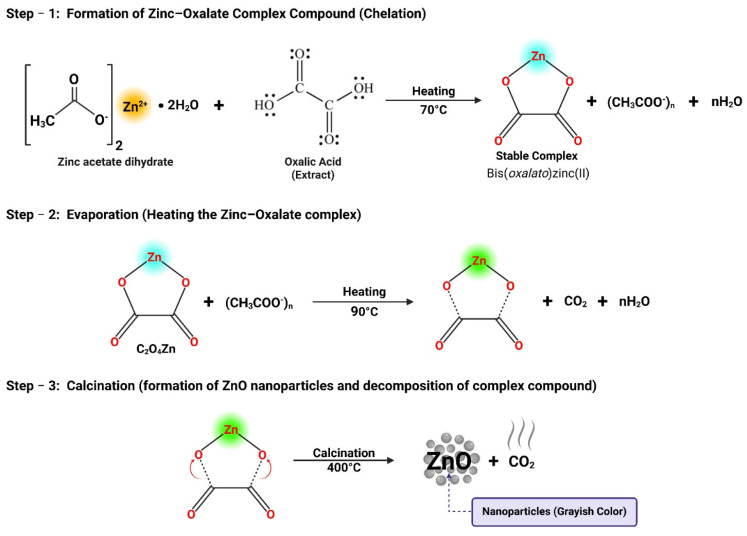
Proposed reaction mechanism for the formation of ZnO using natural extract as precursor. The scheme summarizes the sequential steps involved: chelation of zinc ions to form a zinc–oxalate complex, solvent evaporation upon heating, and subsequent calcination leading to the decomposition of the coordination compound and the formation of ZnO nanoparticles.

These parts of *Agave tequilana* possess unique phytochemical and organic compositions, including oxalic acids, alkaloids, steroids, glycosides, polyphenols, terpenoids, flavonoids, aromatic hydrocarbons, and resins [58,59], which vary significantly in their uses, ranging from economic products to agricultural waste. Such an approach not only expands the understanding of this plant’s untapped potential but also makes it ideal for evaluating structural influence on NPs synthesis, as they facilitate stabilization and reduction reactions. The potential reaction mechanism for the synthesis of ZnO-NPs using natural extracts is illustrated in Figure 2. In this mechanism the oxalic acid derived from the carboxylic acids class of phytochemicals reacts with the precursor salt and binds with Zn^2+^ ions, forming a stable coordination compound bis(oxalate)zinc (II) or zinc oxalate, while acetate ions remain dissolved in the liquid medium, as shown in the following reaction:

Zn(CH_3_COO)_2_·2H_2_O + C_2_H_2_O_4_·nH_2_O → C_2_O_4_Zn + 2CH_3_COO^−^ + 2H_2_O

C_2_O_4_Zn + 2CH_3_COO^−^ + 2H_2_O → ZnO + CO_2_ + nH_2_O

To evaporate the water residue, the solution is heated at 80–90 °C, where partial decomposition takes place, and dried precipitates of zinc oxalate are obtained. Afterwards, thermal decomposition at high temperature breaks the stable coordination compound, yielding the crystallized ZnO-NPs and releasing carbon dioxide (CO_2_) and water (H_2_O) molecules.

Previous studies involving Agave species have contributed to green ZnO synthesis: for example, *Agave americana* extract has been used to prepare ZnO@C nanocomposites with enhanced photocatalytic activity [60], and *Agave tequilana* lignin has been combined with ZnO nanoparticles for skin photoprotection [61]. Reports on other Agave species are few or focus on different nanomaterials or downstream applications. The present study explores the green synthesis of ZnO-NPs using extracts derived from distinct parts of *Agave tequilana*, aiming to establish a comparative understanding of their efficiency and suitability in sustainable nanomaterial production. Considering that each plant part exhibits a unique phytochemical profile, the investigation provides valuable insight into how these differences influence the physicochemical and functional properties of the resulting nanoparticles. This approach underscores the relevance of *Agave tequilana* as an underexplored yet promising resource for sustainable nanotechnology. Synthesized NPs were thoroughly characterized by advanced analytical techniques, including UV-Vis spectroscopy, Fourier-transform infrared spectroscopy (FTIR), X-ray diffraction (XRD), field emission scanning electron microscopy (FESEM), and energy-dispersive X-ray spectroscopy (EDS). These methods offered detailed insights into the morphology and size of the NPs, crystalline structure, band gap, and elemental composition. In addition, the antibacterial efficacy of the NPs was evaluated to determine which plant part produces the most effective ZnO-NPs for potential biomedical use. This comprehensive approach not only reinforces the ecological and functional value of *Agave tequilana* but also underscores its relevance as a sustainable resource in the development of eco-friendly nanomaterials. Ultimately, the findings pave the way for innovative plant-based strategies in antimicrobial research and make a meaningful contribution to advancing green nanotechnology.

## 2. Results

**Figure 3:**
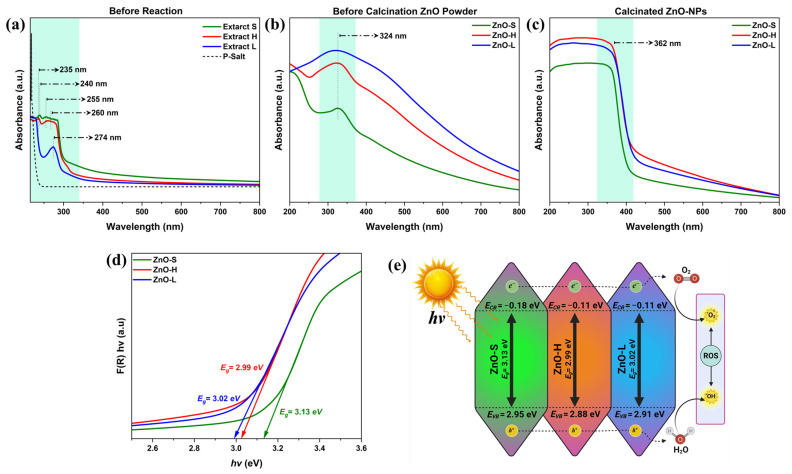
(**a**) UV-Vis spectra of *Agave tequilana* extracts and the precursor salt, (**b**) UV-Vis spectra of ZnO powders before calcination, (**c**) UV-Vis spectra of calcinated ZnO-NPs, (**d**) energies of bandgap, and (**e**) conduction and valence band energy positions of ZnO-S, ZnO-H, and ZnO-L samples. The green-shaded region in spectra (**a**–**c**) highlights the absorption region of the samples.

The UV-Vis spectroscopy study was conducted at three different stages of the synthesis process, namely (1) before the reaction, which refers to the extracts obtained from the parts of the *Agave tequilana* plant and before being mixed with the precursor salt; (2) after the reaction, referring to the dry powders obtained after mixing with the extracts but before subjecting them to a calcination process; and (3) after the powders were calcinated in the furnace. The analysis at these three stages was performed to monitor the formation of ZnO-NPs throughout the different steps of the synthesis from natural extracts of different parts of the *Agave tequilana* plant: the stalk (identified as “S”), the heart (identified as “H”), and the leaves (identified as “L”). Analysis of the optical properties at each stage provided relevant information on the evolution of the synthesized samples from the precursor salt to the production of zinc oxide, allowing for greater insight into what happens in each step of the synthesis and highlighting the importance of each step of the synthesis process, as illustrated in Figure 3.

The initial UV–Vis spectra of the plant extracts demonstrate individual absorption features for the stalk (Extract “S”), heart (Extract “H”), and leaves (Extract “L”) in the range 230–300 nm (see Figure 3a). Extract S exhibits two absorption peaks at 240 nm and 255 nm, Extract H presents an absorption peak at 235 nm, whereas Extract L displays a single intense peak at 274 nm. All these peaks are characteristic of π–π* transitions in aromatic rings, commonly associated with phytochemicals such as flavonoids, tannins, carboxylic acids, and phenolic acids [62,63,64]. These biomolecules are known to play key roles in reducing and stabilizing metal ions during phyto-assisted NP synthesis. The observed variation in absorption maxima and spectral profiles among the extracts suggests differences in secondary metabolite composition throughout the stalk, heart, and leaves of *Agave tequilana*. Notably, Extract L showed a broader, more intense peak at 274 nm, indicating a higher concentration or greater diversity of UV-active phytoconstituents. This observation supports the typically richer metabolite profile commonly found in the plant leaves. In contrast, the spectrum of the precursor salt (“P-Salt”—zinc acetate dihydrate) showed no significant absorption in the 230–300 nm range, indicating the absence of such bioactive organic molecules in the precursor salt. This spectral baseline serves as an essential control, confirming that the UV absorption features observed in the extracts arise purely from plant-derived phytochemicals.

Figure 3b presents the UV–Vis absorption spectra of the synthesized ZnO powders before the calcination phase. All samples exhibit broad absorption bands around 324 nm, symbolic of Zn-containing intermediate species. These absorption features are likely attributed to the formation of zinc oxalate or related zinc–organic complexes [65], arising from interactions between Zn^2+^ ions and phytochemicals in the extracts. Organic acids such as oxalic and malic acids, present in the plant material, likely coordinate with Zn^2+^ to form intermediates like zinc oxalate hydrate (C_2_O_4_Zn·nH_2_O). These complexes typically exhibit ligand-to-metal charge-transfer bands, which account for the observed UV absorption. Notably, ZnO-L exhibits the highest absorbance intensity, followed by ZnO-H and ZnO-S, suggesting possible differences in the concentrations of these intermediates, depending on the extract composition. A broad peak around 324 nm in the ZnO-L spectrum further supports the presence of coordinated zinc–organic species. The spectral profiles suggest that ZnO synthesis initially proceeds via stable zinc–organic intermediates, with zinc oxalate likely serving as a key transitional phase that may decompose upon calcination to yield crystalline ZnO.

Figure 3c illustrates the UV–Vis absorption spectra of calcinated ZnO-NPs. All samples exhibit strong, sharp absorption bands around 362 nm, a region typically associated with electron transitions in ZnO [15,66]. This shift in spectral features, compared with the broader absorption bands observed before calcination, highlights the critical role of the calcination step. Calcination enables the thermal decomposition of zinc oxalate intermediates formed during the initial phyto-assisted green synthesis into ZnO. The disappearance of the broad UV absorptions associated with zinc–organic complexes, along with the emergence of sharp absorption edges, suggests the effective removal of organic constituents and the development of well-defined ZnO structures. The observed variations in absorbance intensity and edge definition among the samples suggest differences in nanoparticle size, potentially arising from the specific phytochemical composition of each extract. Tauc’s equation was employed to estimate the optical band gap (*Eg*) values for ZnO-S, ZnO-H, and ZnO-L samples, as presented in Figure 3d. The calculated *Eg* values were subsequently used to determine the conduction band (*E_CB_*) and valence band (*E_VB_*) positions of the NPs through standard empirical relations. Among the three samples, ZnO-S exhibited the highest optical *Eg* value of 3.13 eV. The corresponding *E_CB_* and *E_VB_* positions were calculated to be −0.18 eV and 2.95 eV, respectively, as illustrated in Figure 3e. This relatively wide band gap indicates efficient UV light absorption, which is a hallmark of small particle sizes where defect states rather than quantum confinement influence the optical transitions [67]. Economically, the stalk of *Agave tequilana* is underutilized, offering a sustainable pathway for the synthesis of high-performance materials.

ZnO-H NPs exhibit an absorption slightly shifted towards longer wavelengths compared to ZnO-S, correlating to an *Eg* of 2.99 eV. The *E_CB_* and *E_VB_* positions are calculated at −0.11 eV and 2.88 eV, respectively. The slightly lower *E_CB_* position compared to ZnO-S enhances the ability of ZnO-H to reduce oxygen molecules into reactive superoxide anions (O_2_^−^). Additionally, the *E_VB_* energy remains high enough to generate hydroxyl radicals, making ZnO-H highly efficient in ROS production under light exposure. These features make ZnO-H a versatile material for photo-assisted applications under both UV and visible light conditions. Although the heart of *Agave tequilana* is best known for its economic value in Tequila production, its use in the green synthesis of nanomaterials offers an alternative pathway to generate added value from the plant, providing a sustainable means of producing multifunctional materials from agricultural resources.

ZnO-L NPs display an *Eg* value of 3.02 eV, with *E_CB_* and *E_VB_* positions at −0.11 eV and 2.91 eV, respectively. The reduced *Eg* enhances visible-light absorption, potentially making ZnO-L more efficient for photocatalytic and antibacterial applications under ambient light. From an economic standpoint, the use of *Agave tequilana* leaves, often considered agricultural waste, is a cost-effective, environmentally friendly method for producing ZnO-NPs. The ability of ZnO-L to leverage visible light for photo-assisted processes further expands its application potential.

**Figure 4:**
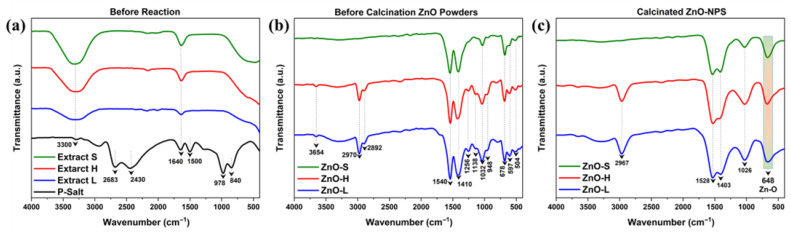
(**a**) FTIR spectra of *Agave tequilana* extracts and precursor salt, (**b**) spectra of ZnO powders before calcination, and (**c**) spectra of calcinated ZnO-S, ZnO-H, and ZnO-L samples.

The FTIR spectra of *Agave tequilana* extracts and the precursor salt are presented in Figure 4a. In the plant extracts, a characteristic absorption band at ~1640 cm^−1^ was observed, which is attributed to the stretching vibrations of carbonyl groups (C=O). This absorption can be associated with the presence of aldehydes, ketones, and flavonoid derivatives, which are well-documented phytoconstituents of Agave species [24]. A broad band centered around ~3300 cm^−1^ corresponds to O–H stretching vibrations of hydroxyl groups, which is typically indicative of alcohols and phenolic compounds. The intensity and width of this signal suggest significant contributions from polyphenols, flavonoids, and saponins, confirming the abundance of hydroxylated phytochemicals in the extracts [68]. The presence of these functional groups is particularly relevant, as they are known to participate in metal ion chelation, reduction, and stabilization, thereby facilitating subsequent nanoparticle formation.

In contrast, the FTIR spectrum of the precursor salt (zinc acetate dihydrate) shows a band at ~840 cm^−1^ that corresponds to skeletal C–H vibrations of the acetate ligand, while the absorption near ~978 cm^−1^ is assigned to C–O stretching vibrations of acetate groups. A distinct feature at ~1500 cm^−1^ reflects CH_3_ deformation coupled with asymmetric stretching of the carboxylate group. In the mid-frequency region, the prominent band at ~1640 cm^−1^ is characteristic of H–O–H bending vibrations, confirming the presence of coordinated crystalline water. Two absorptions at ~2430 cm^−1^ and ~2683 cm^−1^ were observed, corresponding to O–C–O vibrational modes and aliphatic C–H stretching, respectively. Finally, the broad band centered around ~3300 cm^−1^ is attributed to O–H stretching vibrations of water molecules of crystallization [69]. The FTIR spectra of *Agave tequilana* extracts (Stalk, heart, leaves) evidenced the presence of hydroxyl and carbonyl groups, indicative of polyphenols, flavonoids, or related phytochemicals with reducing and stabilizing potential. The precursor salt spectrum, on the other hand, was dominated by acetate- and water-related vibrations, consistent with the structural identity of zinc acetate dihydrate as the Zn^2+^ source.

The FTIR spectra of ZnO powders synthesized using the stalk (ZnO-S), heart (ZnO-H), and leaf (ZnO-L) extracts from *Agave tequilana* before calcination are presented in Figure 4b. Many absorption bands were observed, which reflect both the presence of ZnO lattice vibrations and the organic functional groups originating from the phytochemicals in the plant extracts. Several absorption bands were common across all three samples (ZnO-S, ZnO-H, and ZnO-L). The peaks appearing at 678, 597, and 504 cm^−1^ are characteristic of Zn–O stretching vibrations, indicating the formation of Zn–O bonds [70]. In addition, the absorption at 1032 cm^−1^ is attributed to C–O stretching vibrations of alcohols, esters, or polysaccharides [71]. The peak at 1410 cm^−1^ suggests the presence of organic molecules or functional groups associated with specific chemical bonds. It could be related to C-H bending vibrations in aliphatic compounds or stretching of carboxylate groups (−COO^−^) [72]. A strong band at 1540 cm^−1^ can be assigned to asymmetric carboxylate stretching and/or C=C stretching of the aromatic ring of phytochemicals [71]. The presence of these peaks in all three spectra indicates that similar groups of phytochemicals from different plant parts were present during the formation of ZnO-NPs.

In contrast, some additional absorption features were observed only in ZnO-H and ZnO-L, but were absent in ZnO-S. These include a broad band at 3654 cm^−1^ due to O–H stretching vibrations of hydroxyl groups from alcohols, phenols, or adsorbed water molecules [73]. The peaks at 2970 and 2892 cm^−1^ arise from the aliphatic C–H stretching of –CH_2_ and –CH_3_ groups, suggesting the contribution of terpenoids and fatty acid residues [72]. Moreover, the absorptions at 1256 and 1138 cm^−1^ may be associated with C=O stretching vibrations of carboxylic acid and C–O–C stretching of phenolics, esters, and polysaccharides [74], while the band at 948 cm^−1^ can be associated with C-H bending vibrations in organic compounds, possibly indicating the presence of hydrocarbons or aliphatic groups [72]. The presence of these bands exclusively in ZnO-H and ZnO-L indicates that the heart and leaves of *Agave tequilana* harbor a greater diversity of phytochemicals, which may play an essential role in stabilizing and functionalizing the NPs. Overall, the FTIR results demonstrate that while all three plant parts contributed to NPs formation through similar functional groups, the extracts from the heart and leaves contained additional biomolecules that may enhance the capping and stabilization of ZnO-NPs.

The FTIR spectra of the calcinated ZnO powders derived from stalk (ZnO-S), heart (ZnO-H), and leaves (ZnO-L) are shown in Figure 4c. After calcination, the spectra show fewer absorption bands due to thermal removal of volatile organics and phytochemical residues, leaving mainly ZnO lattice vibrations with minor surface-bound functional groups. An absorption band at ~648 cm^−1^ is observed in all samples and is associated with the Zn–O stretching mode of wurtzite ZnO, confirming the formation of crystalline ZnO after calcination [75]. A weaker band at ~1026 cm^−1^ is associated with C–O stretching vibrations of alcohols, ethers, or polysaccharides [71]. The bands around 1403 cm^−1^ and 1528 cm^−1^ are associated with asymmetric and symmetric stretching of carboxylate groups (−COO^−^) [72], respectively.

Additionally, in the higher wavenumber region, a distinct absorption at ~2967 cm^−1^ is observed in ZnO-H and ZnO-L, corresponding to aliphatic C–H stretching (–CH_2_/–CH_3_ groups) [72]. This band is notably absent in ZnO-S, which indicates a more complete removal of aliphatic organic residues during calcination in the stalk-derived sample. The difference suggests that the heart and leaves of *Agave tequilana* contain relatively higher proportions of aliphatic phytochemicals that remain weakly bound to the ZnO surface even after calcination. Overall, the FTIR spectra after calcination confirm that ZnO lattice vibrations dominate in all samples, while minor carboxylate and aliphatic groups persist, particularly in ZnO-H and ZnO-L, reflecting the influence of plant-part-specific phytochemical composition on NPs stabilization.

**Figure 5:**
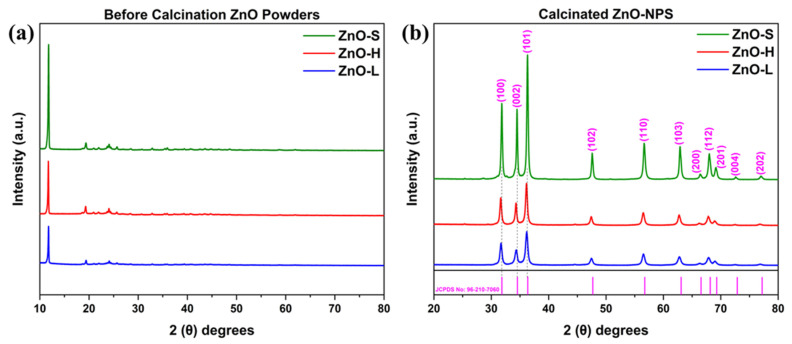
XRD patterns of powders (**a**) before calcination and (**b**) after calcination of ZnO-S, ZnO-H, and ZnO-L samples.

The crystalline structures of ZnO-S, ZnO-H, and ZnO-L solid materials were studied by XRD. The resulting diffractograms before and after powder calcination are shown in Figure 5a,b, respectively. As illustrated in Figure 5a, the XRD patterns of the powders before calcination exhibit mostly low-intensity peaks, indicating that the ZnO precursor complexes formed during synthesis have not yet fully crystallized into ZnO-NPs. This phenomenon is typical of as-synthesized biogenic materials, where organic compounds and phytochemicals from plant extracts often inhibit full crystallization during the initial phase. These organic moieties likely act as stabilizers and may disrupt the long-range atomic ordering. On the other hand, Figure 5b demonstrates visibly different XRD patterns for the calcinated samples ZnO-S, ZnO-H and ZnO-L showing the diffraction peaks at 2θ values of approximately 31.73°, 34.44°, 36.35°, 47.56°, 56.69°, 62.85°, 66.44°, 68.01°, 69.16°, 72.56°, and 77.07°, which correspond, respectively, to the diffraction planes (100), (002), (101), (102), (110), (103), (200), (112), (201), (004), and (202). All peaks match the diffraction pattern of ZnO, according to JCPDS card no. 96-210-7060. No secondary phases or impurity-related peaks were observed, indicating that the calcined products are single-phase crystalline ZnO. ZnO-S exhibited the most intense diffraction peaks, which may imply a higher degree of crystallinity compared to ZnO-H and ZnO-L. The relatively sharper peaks in ZnO-S could be influenced by the unique composition of the stalk extract, which may have affected crystal nucleation and growth during synthesis. In contrast, ZnO-H and ZnO-L showed broader and less intense peaks, which indicate a less crystalline structure.

To further investigate the structural characteristics, the crystallite sizes (*D*) of the ZnO-NPs were calculated using the Debye–Scherrer equation (Equation (1)), using the most intense diffraction peak for each sample. The shape factor (*K*) was set to 0.9, and the X-ray source used was Cu Kα radiation (*λ* = 1.5406 *Å*). The full width at half maximum (FWHM) of the diffraction patterns is established as *β* in radians, along with Bragg’s angle (*θ*), which were used for the calculations of each sample separately to obtain the crystallite size. The crystallinity trend for each sample of ZnO-S, ZnO-H, and ZnO-L were found to be 40.14 nm, 22.56 nm, and 18.46 nm, respectively, which coincides with the trend observed in the XRD diffractograms based on the width of the diffraction peaks.

(1) $$D=\frac{K\cdot \lambda }{\beta \cdot cos\theta }$$

Overall, the XRD analysis shows a clear structural transformation in the ZnO powders upon calcination. Before calcination, the samples exhibited a crystal structure characterized by a high-intensity peak at approximately 12°, which is more closely associated with the precursor salt and is entirely different from that of ZnO. Calcination at 400 °C for 3 h, applied to all samples, showed that this step is crucial for converting the materials to ZnO with high crystallinity, as evidenced by sharp, high-intensity diffraction peaks. The absence of impurity peaks suggests the formation of phase-pure ZnO. Among the samples, ZnO-S showed the smallest crystallite size, followed by ZnO-H and ZnO-L, highlighting the significant influence of the plant part used on the structural quality of the synthesized nanoparticles.

**Figure 6:**
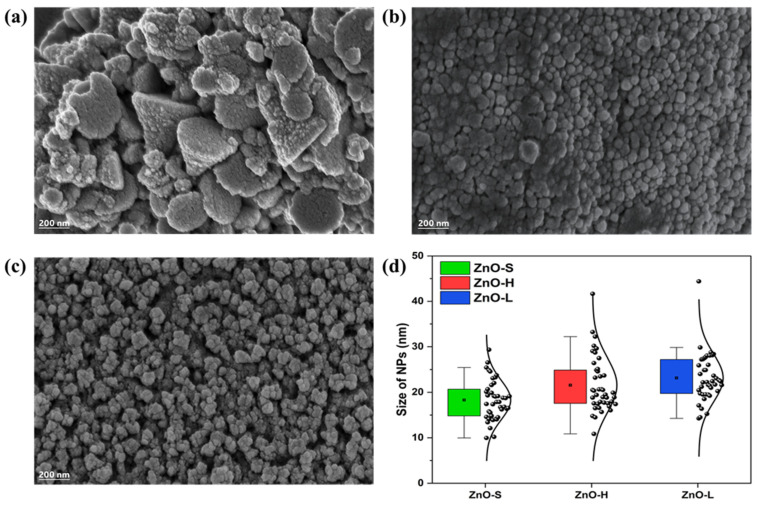
FESEM graphs of the green-synthesized ZnO-NPs (**a**) ZnO-S, (**b**) ZnO-H, and (**c**) ZnO-L showing variation in morphology and particle aggregation. (**d**) Nanoparticle size distribution, where the black markers within each box represent the mean value for each sample, and the black curve shows the normal distribution obtained from the measurements.

The surface morphology and particle distribution of the green-synthesized ZnO-NPs were examined using FESEM. The FESEM images in Figure 6a–c illustrate morphological variations across different plant parts used in the synthesis process. Additionally, Figure 6d provides an overview of the particle size distribution for ZnO-S, ZnO-H, and ZnO-L, offering further comprehension into the structural characteristics of the synthesized NPs.

The FESEM micrography of Figure 6a shows the surface morphologies of calcinated ZnO-S NPs synthesized using the stalk extract of *Agave tequilana*. The image reveals that the NPs are highly agglomerated, forming irregular clusters with a notable presence of pyramid-shaped structures. These clusters appear to consist of smaller, well-defined primary particles with distinguishable granular structures. The morphology suggests uniform particle shape, with mostly spherical or near-spherical outlines. Figure 6b shows the morphology of calcined ZnO-H nanoparticles synthesized from the heart extract, displaying densely packed, predominantly spherical nanostructures that form a homogeneous, compact layer with minimal shape variation. The particle morphology of calcinated ZnO-L NPs synthesized using the leaves extract can be observed in Figure 6c. These NPs also show agglomerates comprising predominantly spherical or near-spherical particles, but they do not show the same compaction as that observed in the ZnO-H sample. FESEM analysis of ZnO-S, ZnO-H, and ZnO-L revealed distinct yet comparable morphological characteristics, demonstrating the influence of the different parts of *Agave tequilana* (stalk, heart, and leaves) on ZnO-NP synthesis. All samples exposed agglomerated structures composed of predominantly spherical NPs with a high degree of uniformity. The particle size distribution of these ZnO-NPs was determined using ImageJ 1.54g software. The results, presented in the box plot of Figure 6d, reveal distinct average particle sizes and distributions for each sample, indicating that the precursor material influences NP size. The ZnO-S sample exhibited the smallest average particle size of 18.31 nm. The narrow distribution in the box plot indicates a high degree of uniformity, suggesting that the stalk precursor promoted the formation of smaller, more uniform NPs. This may be attributed to the specific biomolecules in the stalk that effectively stabilize nuclei during NPs formation. In contrast, the ZnO-H sample showed a slightly larger average particle size of 21.51 nm. The broader size distribution observed for ZnO-H shows a moderate increase compared to ZnO-S. The ZnO-L sample demonstrated the largest average particle size of 23.16 nm among the three samples.

**Figure 7:**
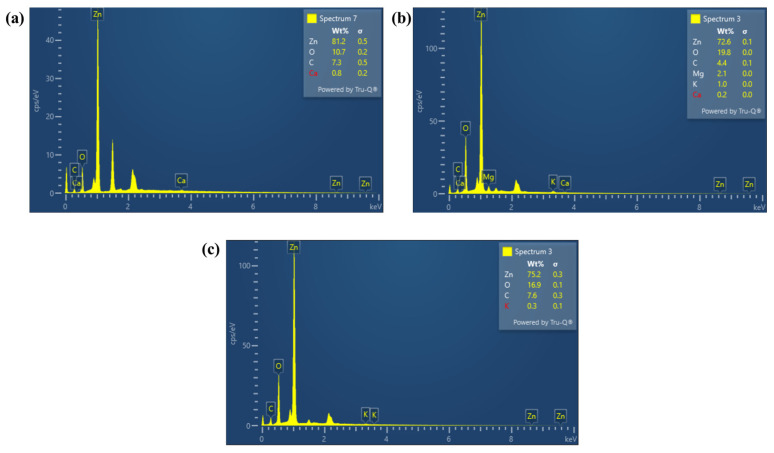
EDS spectra of the ZnO-NPs (**a**) ZnO-S, (**b**) ZnO-H, and (**c**) ZnO-L showing the elemental composition of the three samples.

The EDS analysis was employed to confirm the elemental composition and purity of the synthesized ZnO-NPs. EDS spectra for ZnO-S, ZnO-H, and ZnO-L presented in Figure 7a–c validate prominent peaks corresponding to zinc (Zn) and oxygen (O), as the primary elements of the analyzed materials. For ZnO-S the elemental composition by weight revealed a high Zn content of 81.2%, accompanied by 10.7% O, along with trace amounts of carbon (C, 7.3%) and calcium (Ca, 0.8%). Two clearly visible signals in the EDS spectrum of the ZnO-S sample have not been labeled or accounted for in the elemental analysis, since they correspond to aluminum (≈1.5 keV) and gold (≈2.1 keV) signals, coming from the pin where the sample was dispersed and the conductive coating added during the sample preparation, respectively. ZnO-H showed a Zn content of 72.6% and a relatively higher O percentage of 19.8%, along with small quantities of C (4.4%), magnesium (Mg, 2.1%), potassium (K, 1.0%), and Ca (0.2%). The slightly lower Zn and elevated O content, compared to ZnO-S, may suggest the presence of more oxygen-containing phytochemicals in the heart extract or a subtle variation in oxidation during synthesis. The detection of elements such as Mg and K is likely derived from natural minerals present in the plant extract [58,76]. In the case of ZnO-L, Zn is illustrated for 75.2% by weight, with O at 16.9% and C at 7.6%, along with a small amount of K (0.3%). The presence of K may stem from the intrinsic mineral content of the leaves extract [76]. The EDS results confirm the formation of zinc oxide, with Zn and O atomic ratios close to 1:1 in all three samples (ZnO-S, ZnO-H, and ZnO-L), indicating successful ZnO synthesis from each extract. Minor impurities such as C, Ca, Mg, and K are expected outcomes of green synthesis approaches using natural plant extracts. The comprehensive physicochemical characterizations confirm that the green synthesis approach using various parts of the *Agave tequilana* plant effectively yields ZnO-NPs with interesting features, including nanoscale size, well-defined morphology, and high crystallinity. Building upon these promising characteristics, the subsequent section explores the antibacterial activity of the synthesized NPs, assessing their practical relevance in biomedical applications.

### Antibacterial Activity

**Figure 8:**
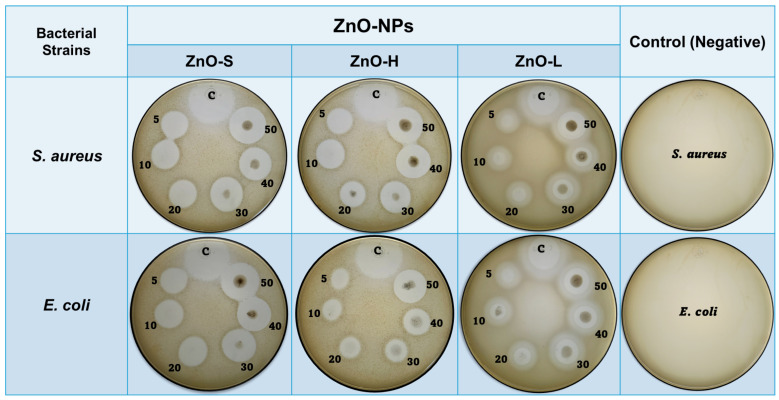
Inhibition zones demonstrating the antibacterial activity of green-synthesized ZnO-NPs against the *S. aureus* and *E. coli*. The numbers shown in each image correspond to the sample concentrations in µg/mL, and the letter “C” denotes the positive control (kanamycin).

The antibacterial potential of the green-synthesized ZnO-NPs was systematically evaluated using the agar well diffusion method against *Staphylococcus aureus* (Gram-positive) and *Escherichia coli* (Gram-negative). The inhibition zone (IZ) was measured after 24 h of incubation at 37 °C, and the results are summarized in Table 2. Kanamycin, used as positive control, produced clear IZ against both *Staphylococcus aureus* and *Escherichia coli*, confirming the sensitivity of the assay (see Figure 8). In contrast, the negative control exhibited no antibacterial effect. All the green-synthesized ZnO-NPs demonstrated dose-dependent antibacterial activity against both bacterial strains, with the highest bactericidal response at the highest concentration (50 μg/mL). The pronounced bactericidal performance observed across these concentrations highlights the effective antimicrobial potential of ZnO-NPs synthesized using different parts of the *Agave tequilana* plant. These findings underscore the untapped potential of Agave as a sustainable source for producing bio-functional nanomaterials with promising applications in antimicrobial therapies.

**Figure 9:**
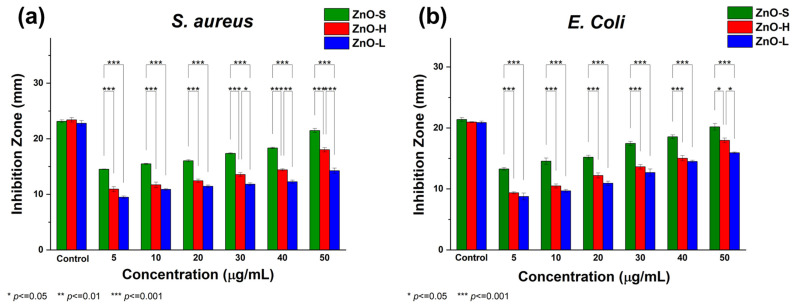
Antibacterial activity of ZnO-S, ZnO-H, and ZnO-L against (**a**) *S. aureus* and (**b**) *E. coli*, evaluated across varying concentrations of ZnO-NPs, demonstrating a clear dose-dependent response of ZnO-NPs. Interestingly, at the highest concentration tested (50 μg/mL), the ZnO-S sample exhibited an effect comparable to that of kanamycin. Results are expressed as mean ± standard deviation (SD) for six concentrations and the positive control (kanamycin). * *p* < 0.05, ** *p* < 0.01, and *** *p* < 0.001.

A graphical comparison with statistical analysis of antimicrobial activity results is presented in Figure 9. Among the three samples studied in this work, ZnO-S showed the most pronounced antibacterial activity, particularly at higher concentrations. At 50 µg/mL, it produced an inhibition zone of 21.49 ± 0.52 mm against *S. aureus* and 20.18 ± 0.76 mm against *E. coli*, closely approaching the standard antibiotic Kanamycin (23.14 ± 0.38 mm and 22.53 ± 0.30 mm, respectively). This superior performance can be attributed to the smallest average particle size (~18.31 nm), which increases the surface-to-volume ratio and enhances interaction with bacterial membranes. Importantly, the stalk of the *Agave tequilana* plant is typically discarded as agricultural waste; hence, utilizing this part for NPs synthesis not only contributes to waste reduction but also adds economic value to an otherwise underutilized resource, making ZnO-S a sustainable and cost-effective candidate for large-scale production of nanomaterials with antibacterial performance. The mechanism of action for ZnO-S may involve multiple pathways: (i) generation of ROS such as hydrogen peroxide, hydroxyl radicals, and superoxide anions that damage cellular components; (ii) direct attachment to the bacterial membrane, causing mechanical disruption and leakage of intracellular contents; and (iii) Zn^2+^ ion release, which interferes with bacterial enzyme activity and protein synthesis. The smaller particle size of ZnO-S may facilitate deeper penetration into bacterial cells, thereby enhancing its cytotoxic effects, especially against Gram-positive *S. aureus*, which lacks the protective outer membrane of Gram-negative bacteria.

ZnO-H, synthesized from the heart of the *Agave tequilana* plant, the core commercially utilized in Tequila production, exhibited moderate yet significant antibacterial activity. At the highest concentration, it showed an inhibition zone of 18.02 ± 0.55 mm for *S. aureus* and 17.96 ± 0.53 mm for *E. coli*. While it is less effective than ZnO-S, its performance still underscores the bioactivity of NPs derived from economically valuable plant extracts. The moderate particle size (~21.57 nm) of ZnO-H suggests a slightly reduced surface reactivity compared to ZnO-S, possibly due to the difference in particle sizes. ZnO-L, synthesized from the leaves extract, a commonly discarded agricultural residue, demonstrated the least antibacterial activity, although still notable. At 50 µg/mL, it achieved IZs of 14.24 ± 0.68 mm for *S. aureus* and 15.92 ± 0.19 mm for *E. coli*. This relatively lower performance can be associated with its larger particle size (~23.16 nm). Nevertheless, the activity of ZnO-L remains relevant, particularly considering that it is synthesized from a plant part that is typically regarded as waste. Interestingly, all ZnO-NPs samples showed greater sensitivity toward *S. aureus* than *E. coli* at lower concentrations, which aligns with the known structural differences in bacterial cell walls. *E. coli*, as a Gram-negative bacterium, possesses an additional lipopolysaccharide outer membrane that offers enhanced protection. However, at higher ZnO-NPs concentrations, this resistance was overcome, as seen by the significantly increased inhibition zones.

## 3. Discussion

The use of nanomaterials has become increasingly relevant in medical and biotechnological fields, particularly in the search for new antimicrobial agents capable of addressing the growing challenge of antimicrobial resistance. To meet these demands, it is essential to develop synthesis routes that ensure not only precise control over material properties but also environmental responsibility. Conventional chemical synthesis methods often rely on toxic reagents, posing risks to both human health and the environment. In contrast, green synthesis offers a sustainable and safer alternative, aligning with the principles of green chemistry by minimizing hazardous by-products and employing renewable resources.

In this context, the present study demonstrates the successful green synthesis of ZnO nanoparticles (ZnO-NPs) using extracts from three different parts of the *Agave tequilana* plant: the heart, leaves, and stalk. The phytochemicals naturally present in these extracts act as reducing and stabilizing agents, enabling the formation of ZnO nanoparticles without the use of harmful chemicals. Characterization results confirm the efficient conversion of the precursor salt into ZnO with average particle sizes below 25 nm, validating the potential of *Agave tequilana* as a sustainable bioresource for nanomaterial production.

Calcination proved essential for defining the final nanoparticle morphology and crystal structure, enhancing crystallinity, and improving uniformity by removing residual organics. The combined influence of precursor composition and calcination conditions underscores the importance of controlling synthesis parameters to tune the physicochemical properties of ZnO-NPs for specific biomedical purposes.

Among the samples, ZnO-S synthesized from the *Agave tequilana* stalk extract exhibited the most promising results, highlighting the exceptional potential of this plant waste as a precursor for high-performance nanomaterials. The stalk and leaves, typically discarded during agave processing, were found to produce nanoparticles with suitable size and morphology for antimicrobial applications, transforming low-value agricultural residues into valuable raw materials for the synthesis of high-performance nanomaterials. This outcome represents a clear opportunity to apply circular economy principles to materials science, where waste valorization converges with sustainable innovation.

Moreover, the antimicrobial performance of the synthesized ZnO-NPs reinforces their potential as bioactive materials for the development of next-generation antimicrobial agents. By enabling the sustainable production of ZnO-NPs with strong bactericidal potential, this work demonstrates a viable route toward environmentally responsible nanomaterials that can help counteract antimicrobial resistance. Overall, the findings emphasize that the combination of green synthesis and waste valorization not only reduces environmental impact but also opens new pathways for designing functional materials with direct relevance to healthcare and biotechnology.

The antibacterial activity of ZnO-NPs is generally attributed to three interconnected mechanisms: the generation of ROS, the release of Zn^2+^ ions, and direct interactions between the nanoparticles and the bacterial cell membrane. Numerous studies have shown that ROS production is strongly dependent on particle size. Smaller ZnO-NPs exhibit a higher surface-to-volume ratio, which promotes greater oxygen adsorption and more efficient electron–hole separation, ultimately enhancing ROS formation [77,78]. Particle size also affects the optical bandgap, as a reduction in crystallite size slightly widens the bandgap, facilitating increased ROS generation under ambient conditions [79,80]. These mechanistic considerations are consistent with our findings, as the smallest ZnO-NPs (stalk-derived) displayed the highest antibacterial activity.

## 4. Materials and Methods

### 4.1. Chemicals

Precursor salt Zinc-acetate-dihydrate [Zn(CH_3_COO)_2_·2H_2_O], with a reagent-grade purity of ≥98%, was purchased from Sigma-Aldrich and employed directly in the experimental procedures without any subsequent purification steps.

### 4.2. Collection of Plants (Agave tequilana)

Plant samples were carefully collected from their native habitats in full accordance with national and international regulatory standards, following the guidelines established by the IUCN Species Survival Commission (SSC-2001) [81]. The *Agave tequilana* plant and its parts were systematically collected from agricultural fields in the city of Tequila, located in the state of Jalisco, central Mexico (Latitude 32°88′32, Longitude 103°81′65″ W). This region is globally recognized as the heart of Tequila production in Mexico. The collected plant parts intended for extract preparation were thoroughly washed, first with tap water and then with deionized water, to remove any residual dust and soil particles. After washing, the samples were air-dried at room temperature (25 °C) to ensure proper dehydration. The stalk, heart, and leaves were subsequently chopped into small pieces to obtain the extract, and the remaining parts were labeled and carefully stored in polyethylene bags at −20 °C to preserve their quality for future experimentation.

### 4.3. Green Synthesis of ZnO-NPs Using Extracts of Different Parts of Agave tequilana

**Figure 10:**
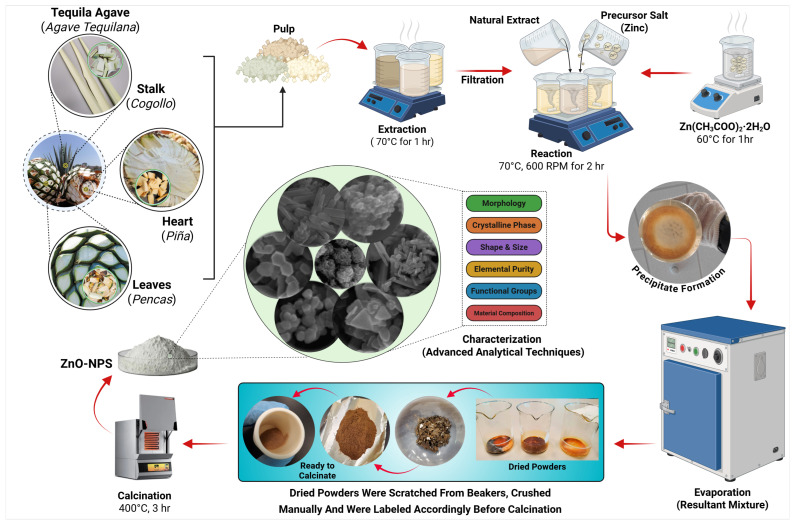
Schematic representation of the green synthesis process used to obtain ZnO nanoparticles from natural extracts derived from different parts of the *Agave tequilana* plant. The diagram illustrates the preparation of the extracts, their reaction with the zinc precursor, and the subsequent steps leading to the formation of ZnO-NPs.

A 150 mL solution of the precursor salt was mixed individually with 75 mL of each prepared extract from the stalk, heart, and leaves of the *Agave tequilana* plant. Reaction conditions were optimized for each mixture and were set at 600 rpm stirring and 70 °C heating for a total of 120 min. As the reaction progressed in aqueous solutions, precipitation formed, and the color shifted from ivory cream to dark brown, forming a colloidal paste. This paste was then placed in a hot-air oven (Memmert GmbH, Schwabach, Germany) at 80–90 °C for 4 h to evaporate any residual water molecules. At this point, a dried powdered sample was obtained. To achieve a finer, more uniform texture, the powder was ground in a mortar with a pestle. To further remove organic residues and obtain a crystalline product, the powders were calcined at 400 °C for 3 h. As a result, the calcinated ZnO-NPs exhibited a dark gray color and were marked as the final product. ZnO-NPs were stored in airtight containers at room temperature, avoiding sunlight for subsequent advanced analytical characterization. The entire green synthesis method is schematically illustrated in Figure 10.

### 4.4. Materials Characterization

A noticeable color change was observed when the precursor salt reacted with natural extracts of stalk, heart, and leaves. This shift in colors strongly indicates that a redox reaction was initiated between the extracts and the precursor salt. Various advanced characterization techniques, including UV–Vis, ATR–FTIR, XRD, FESEM, and EDS, were used to confirm the presence of ZnO-NPs and to thoroughly examine their properties. These techniques enabled the evaluation of the optical properties, identification of functional groups, analysis of particle morphology, assessment of crystalline structure, and determination of elemental composition of materials. The Cary-5000 UV–Vis-NIR spectrometer (Agilent Technologies, Santa Clara, CA, USA) was utilized to monitor the absorbance of ZnO-NPs, with measurements performed across a wavelength range of 200–800 nanometers (nm). The instrument was equipped with a poly-tetrafluoro-ethylene (PTFE) integration sphere. Fourier-transform infrared spectroscopy (FTIR) equipped with an attenuated total reflection (ATR) accessory was utilized to evaluate the presence of organic matter and phytochemicals possibly responsible for the reduction and stabilization of ZnO-NPs. The ATR–FTIR spectra were recorded on an IR Affinity-1S spectrometer (Shimadzu, Kyoto, Japan) over the wavenumber range of 400–4000 cm^−1^. X-ray diffraction (XRD) analysis was performed using a PANalytical 600 Powder X-ray Diffractometer (Malvern, United Kingdom). The measurements were conducted at a source voltage of 30 kV, utilizing a Cu Kα radiation source with a wavelength of 1.54 Å. The diffraction patterns were recorded over a range of 8° to 80°, with a step size of 0.05° and a scanning rate of 2°/min. Field-emission scanning electron microscopy (FESEM) imaging was conducted using a Zeiss GeminiSEM 560 (Jena, Germany) equipped with an Oxford detector, operated at 30 kV. Additionally, energy-dispersive X-ray spectroscopy (EDS) was performed using an EVO MA25 scanning electron microscope (Zeiss, Jena, Germany) at 30 kV. For FESEM and EDS analysis, the powder samples were dispersed onto aluminum pins and coated with a layer of gold.

### 4.5. Antibacterial Activity Assessment

The antibacterial performance of the synthesized ZnO-NPs was evaluated against two representative bacterial strains: *Escherichia coli* (ATCC 11229, Gram-negative) and *Staphylococcus aureus* (ATCC 6538, Gram-positive). The bacterial cultures were prepared by growing the strains in nutrient broth at 37 °C for 24 h. For the antimicrobial assay, nutrient agar Petri dishes were prepared using a double-layer method. The base layer consisted of solidified nutrient agar, while the overlay was obtained by mixing 10 mL of nutrient agar (7.5 g/L) with 100 µL of an overnight-grown bacterial culture. After solidification, 5 µL drops of ZnO-NP solutions at concentrations of 5, 10, 20, 30, 40, and 50 µg/mL were placed on the agar surface. Kanamycin (5 µL) was used as a positive control, and sterile 50% glycerol served as the negative control. Petri dishes were incubated at 37 °C for 48 h. Antibacterial efficiency was determined by measuring the diameter of the inhibition zones (mm). All experiments were conducted in triplicate to ensure reproducibility. Experimental data were statistically analyzed using ANOVA followed by Tukey’s HSD post hoc test at a 95% confidence level. Statistical significance was indicated by asterisks according to the *p*-value: * *p* ≤ 0.05, ** *p* ≤ 0.01, *** *p* ≤ 0.001.

## 5. Conclusions

This study highlights the green synthesis of ZnO-NPs using extracts from distinct parts of the *Agave tequilana* plant: the stalk, heart, and leaves. Each component exhibited a significantly different influence on the properties of the synthesized NPs. ZnO-S, derived from stalk extracts, exhibited the smallest crystallite and particle sizes, with the highest antibacterial efficacy against *Staphylococcus aureus* and *Escherichia coli*. ZnO-H and ZnO-L, synthesized from heart and leaves extracts, respectively, also demonstrated antibacterial activity but with lower efficacy than ZnO-S. In the phyto-assisted synthesis reported here, the calcination step proved crucial, transforming the precursors into highly crystalline ZnO-NPs, besides assisting in the removal of organic residues from the natural extracts. A key insight from this work is the crucial role played by plant-derived phytochemicals in the synthesis process. These compounds act as natural reducing and stabilizing agents, facilitating the formation of NPs with desirable characteristics. Future studies should focus on identifying and isolating specific phytochemicals responsible for these effects. Such investigations could deepen our understanding of their role in NPs stabilization, reduction processes, and potential alterations in particle properties.

The findings underscore the untapped potential of agricultural waste, such as stalks and leaves, which are traditionally discarded despite being valuable resources for producing high-performance nanomaterials. This approach not only mitigates environmental impact but also aligns with sustainable practices, promoting the valorization of agricultural residues. The results further establish *Agave tequilana* as a promising candidate for eco-friendly and cost-effective nanotechnology applications.

In conclusion, this research not only advances green nanotechnology by harnessing the inherent potential of *Agave tequilana* but also opens pathways for discovering innovative, sustainable solutions in the biomedical and environmental sectors. By converting agricultural waste into value-added materials, this study exemplifies the power of interdisciplinary science to drive eco-innovative breakthroughs, paving the way for future developments in eco-friendly nanomaterials with multifaceted applications.

## Figures and Tables

**Table 1:** A summary and comparison of the phyto-assisted synthesis of ZnO-NPs using natural extracts of different plants, showing (as the main contribution of each work) the size and shape of the particle obtained, as well as the application assessed.

Plant Source	Part Used	Shape of NPs	Size (nm)	Application	Reference
Lemongrass (*Cymbopogon olivieri*)	Leaves	Spherical	28	Antimicrobial and Anticancer Activity	[32]
Rain of Gold (*Thryallis glauca*)	Leaves	Hexagonal Wurtzite	50	Antioxidants and Antibacterial Activity	[33]
Veldt Grape (*Cissus quadrangularis*)	Stem	Spherical	75–90	Antibacterial and Anticancer Activity	[34]
Golden Shower Tree (*Cassia fistula*)	Leaves	Spherical	68	Antibacterial Activity	[35]
Lemon (*Citrus limon*)	Fruit	Cuboid, Hexagonal Prism, Thin Rods	60.8	Antibacterial and Antihemolytic Activity	[36]
Pennyroyal (*Mentha pulegium*)	Leaves	Semi Spherical	40	Antimicrobial Activity	[37]
Cauliflower (*Brassica Var. botrytis*)	Leaves	Flower Like	52	Antimicrobial Larvicidal Activity	[38]
Tasmanian Blue Gum (*Eucalyptus globules*)	Leaves	Spherical	52–70	Antifungal Activity	[39]
Syrian Mesquite (*Prosopis farcta*)	Aerial	Hexagonal	40–80	Antifungal and Breast Cancer (MCF-7) Activity	[40]
Sandalwood (*Santalum album*)	Leaves	Nanorods	100	Brest Cancer (MCF-7) Activity	[41]
Caper Bush (*C. spinosa* L.)	Fruit	Spherical	37.49	Antioxidant Activity	[42]
Garden Cress (*Lepidium sativum*)	Seeds	Spherical	37–45	Anticancer Activity	[43]
Gangotra (*Cyathocline purpurea*)	Leaves	Spherical	80–120	Antimicrobial Activity	[44]
Maddu Toppu (*Justicia wynaadensis*)	Leaves	Hexagonal Wurtzite	39	Antimitotic and DNA-Binding	[45]
Arrowleaf Sida (*Sida rhombifolia Linn*)	Leaves	Spherical	30.23	Genotoxic and Antibacterial Activity	[46]
White Passionflower (*Passiflora subpeltata*)	Leaves	Hexagonal	45–50	Antibacterial Activity	[47]
Cape Leadwort (*Plumbago auriculata*)	Aerial	Hexagonal	38.3	Antiviral Activity	[48]
Radish (*Raphanus sativus*)	Leaves	Spherical/Hexagonal	66.47	Breast Cancer Cells Antibacterial Activity	[49]
Black Nightshade (*Solanum nigrum*)	Leaves	Quasi-Spherical	30	Anticancer Activity	[50]
Bay Laurel (*Laurus nobilis*)	Leaves	Flower	47.27	Antibacterial Activity	[51]
Sweet Leaf (*Stevia*)	Leaves	Rectangular	50	Antimicrobial Wound-healing Bandages	[52]
Horseradish Tree (*Moringa oleifera*)	Leaves	Spherical	52.24	Antibacterial and Antioxidant Activity	[53]
Roselle (*Hibiscus subdariffa*)	Leaves	Dumbbell	190	Antibacterial and Antidiabetic Activity	[54]

**Table 2:** Antibacterial efficacy of ZnO-S, ZnO-H, and ZnO-L. Comparative analysis of inhibition zones across different concentrations against selected bacterial strains, reported as the average of triplicate measurements (*n* = 3).

Nanomaterials Concentrations (μg/mL)	Zone of Inhibition (mm)					
	ZnO-S		ZnO-H		ZnO-L	
	*S. aureus*	*E. coli*	*S. aureus*	*E. coli*	*S. aureus*	*E. coli*
**5**	14.52 ± 0.06	13.27 ± 0.30	10.93 ± 0.64	9.37 ± 0.23	9.48 ± 0.31	8.76 ± 0.78
**10**	15.48 ± 0.13	14.55 ± 0.73	11.70 ± 0.68	10.48 ± 0.47	10.88 ± 0.21	9.69 ± 0.32
**20**	16.05 ± 0.27	15.21 ± 0.44	12.44 ± 0.43	12.21 ± 0.59	11.44 ± 0.30	10.92 ± 0.50
**30**	17.37 ± 0.17	17.45 ± 0.48	13.57 ± 0.45	13.62 ± 0.53	11.83 ± 0.35	12.69 ± 0.82
**40**	18.34 ± 0.18	18.55 ± 0.46	14.41 ± 0.26	15.03 ± 0.61	12.27 ± 0.39	14.50 ± 0.27
**50**	21.49 ± 0.52	20.18 ± 0.76	18.02 ± 0.55	17.96 ± 0.53	14.24 ± 0.68	15.92 ± 0.19
**Control** **(*Kanamycin*)**	23.14 ± 0.38	22.53 ± 0.30	23.40 ± 0.56	19.19 ± 0.72	22.78 ± 0.69	17.87 ± 0.69

## Data Availability

Data are contained within the article.

## Abbreviations

The following abbreviations are used in this manuscript:

AMR	Anti-Microbial Resistance
ZnO	Zinc Oxide
NPs	Nanoparticles
ZnO-NPs	Zinc Oxide Nanoparticles
MO	Metal Oxide
MO-NPs	Metal Oxide Nanoparticles
ZnO-S	Zinc Oxide Stalk
ZnO-H	Zinc Oxide Heart
ZnO-L	Zinc Oxide Leaves
UV	Ultraviolet
XRD	X-Ray Diffraction
ATR	Attenuated Total Reflectance
FTIR	Fourier-Transform Infrared Spectroscopy
FESEM	Field Emission Scanning Electron Microscopy
EDS	Energy-Dispersive X-Ray Spectroscopy
ROS	Reactive Oxygen Species
mL	Milliliter
g	Gram
mg	Milligram
µg	Microgram
Eg	Bandgap
eV	Electron Volt
NIR	Near-Infrared
nm	Nanometer
mm	Millimeter
cm	Centimeter
L	Liter
IZ	Inhibition Zones
FWHM	Full Width at Half Maximum
JCPDS	Joint Committee on Powder Diffraction Standards
